# Expression of DNA repair genes and its relevance for DNA repair in peripheral immune cells of patients with posttraumatic stress disorder

**DOI:** 10.1038/s41598-022-22001-w

**Published:** 2022-11-04

**Authors:** Alexander Behnke, Matthias Mack, Judy Fieres, Markus Christmann, Alexander Bürkle, María Moreno-Villanueva, Iris-Tatjana Kolassa

**Affiliations:** 1 Clinical and Biological Psychology, Institute of Psychology and Education, Ulm University, Albert-Einstein-Allee 47, 89081 Ulm, Germany; 2 Molecular Toxicology, Department of Biology, University of Konstanz, 78457 Constance, Germany; 3 Applied Toxicology, Institute of Toxicology, University of Mainz, 55131 Mainz, Germany; 4 Department of Sport Science, Human Performance Research Centre, University of Konstanz, 78457 Constance, Germany; 5 Centre of Excellence for Psychotraumatology, Clinical Psychology and Neuropsychology, University of Konstanz, 78464 Constance, Germany

**Keywords:** Molecular biology, Psychology

## Abstract

Posttraumatic stress disorder (PTSD) involves elevated levels of cellular oxidative stress which jeopardizes the integrity of essential cell compartments. Previously, we demonstrated higher levels of DNA lesions in peripheral blood mononuclear cells (PBMCs) in PTSD. Retaining vital levels of DNA integrity requires cells to mobilize compensatory efforts in elevating their DNA-repair capacity. Accordingly, we hypothesized to find increased expression rates of the DNA-repair genes *X-ray repair cross complementing 1* (*XRCC1*), *poly (ADP-ribose) polymerase 1* (*PARP1*), and *polymerase β* (*Polβ*) in PBMCs of PTSD patients as compared to controls, leading to functionally relevant changes in DNA-repair kinetics. In a cohort of 14 refugees with PTSD and 15 without PTSD, we found significantly higher *XRCC1* expression in PTSD patients than controls (*U* = 161.0, *p* = 0.009, Cohen’s *r* = 0.49), and positive correlations between the severity of PTSD symptoms and the expression of *XRCC1* (*r*_S_ = 0.57, *p* = 0.002) and *PARP1* (*r*_S_ = 0.43, *p* = 0.022). Higher *XRCC1* (*F* = 2.39, *p* = 0.010, η^2^_p_ = 0.10) and *PARP1* (*F* = 2.15, *p* = 0.022, η^2^_p_ = 0.09) expression accounted for slower repair of experimentally X-ray irradiation-induced DNA damage, highlighting the possible physiological relevance of altered DNA-repair gene expression in PTSD. Our study provides first evidence for a compensatory regulation of DNA-repair mechanisms in PTSD. We discuss the implications of increased DNA damage and altered DNA-repair mechanisms in immune senescence, premature aging, and increased physical morbidity in PTSD.

## Introduction

Posttraumatic stress disorder (PTSD) involves increased rates of somatic alterations, including dysregulated immunity, neuroendocrine dysregulation, metabolic syndrome, and cardiovascular morbidity^1,2^. Considering its somatic manifestation, PTSD is being increasingly viewed as a systemic illness with both cognitive-behavioral and physiological symptoms^1,3,4^. Recurrent exposure to traumatic stress is postulated to alter the physiological stress response through dysregulating the hypothalamic–pituitary–adrenal (HPA) axis and the autonomic nervous system in PTSD^2,5^. Blunted activity of the HPA axis and parasympathetic nervous system along with increased activity of the sympathetic nervous system contribute to chronic low-grade inflammatory activity^2,5^. Correspondingly, several meta-analyses confirmed elevated levels of peripheral proinflammatory cytokines in PTSD patients^6,7^.

These cytokines were also shown to increase the oxidative stress level in several cell types, including circulating immune cells, i.e., peripheral blood mononuclear cells (PBMCs)^8^. While elevated levels of oxidative stress are meta-analytically established in other stress-associated mental disorder such as major depressive disorder (MDD)^9–11^, few studies have investigated markers of oxidative stress and related cellular damage in PTSD^12–14^. Persistently elevated levels of oxidative stress cause accumulating macromolecular damage to vital cell components, including cell membranes, mitochondria, and the DNA^15–18^.

In our previous study, we investigated the macromolecular integrity of the entire DNA through determining the amount of DNA strand breaks in PBMCs of PTSD patients and controls. We found that PBMCs of PTSD patients exhibited higher amounts of DNA strand breaks than those of controls, which is presumably the consequence of elevated oxidative stress levels in PTSD^19^. At the same time, we observed that the cells’ capacity to repair ex vivo induced DNA damage was not compromised^19^, which indicates that the cells retained the capacity to restore genome integrity.

However, in the case of PTSD, to compensate for the higher DNA damage that is continuously provoked by elevated levels of oxidative stress, cells need to increase their metabolic expenditure for DNA maintenance and repair. Oxidative DNA lesions as well as DNA single-strand breaks (SSB) are remedied by base-excision repair (BER) that is facilitated by various proteins acting together as a co-regulated cascade^20^. DNA-SSB are detected by poly (ADP-ribose) polymerase 1 (PARP1), and via consumption of nicotinamide adenine nucleotide (NAD^+^) a polymeric adenosine diphosphate-ribose chain is produced to mobilize other DNA-repair proteins^21,22^. One of those targets is the scaffolding protein X-ray repair cross-complementing protein 1 (XRCC1) which by itself orchestrates further repair proteins such as Polymerase β (Polβ)^23,24^.

Under chronic oxidative stress, more DNA damage constantly occurs, and hence more DNA repair is to be performed on an ongoing basis. Mobilizing higher repair efforts requires constant supply with DNA-repair proteins. Therefore, we expected to find higher gene expression of *PARP1*, *XRCC1*, and *Polβ* amongst individuals with PTSD. To ascertain whether PTSD-related alterations in the expression of DNA-repair genes are of physiological relevance, we tested whether altered expression of DNA-repair genes accounts for differences in DNA-repair kinetics after ex vivo induction of DNA-SSB through X-ray irradiation.

## Materials/subjects and methods

### Participants and study design

PTSD patients and controls were recruited at the Centre of Excellence for Psychotraumatology (CEP, University of Konstanz, Germany) as part of a larger research project which served to evaluate the effectiveness of Narrative Exposure Therapy among refugees^19,25^. For the present study, sufficient amounts of blood specimens were available from 15 individuals with PTSD and 14 controls. PTSD patients were refugees from different regions of the world (*n* = 7 West Africa, 4 Near East, 2 Central Asia, 1 Balkans). Controls without PTSD were refugees or immigrants from matched regions of the world (*n* = 5 West Africa, 6 Near East, 3 North and East Africa). Majority of PTSD patients had been traumatized by war and torture at their country of origin as well as during displacement and flight (Table 1).

**Table 1:** Characteristics of the study cohort.

	**PTSD (*n* = 14)**	**Control (*n* = 15)**	**Statistic**	***p***
Age (years)	24.5 (8.5) [17, 36]	22.5 (12.7) [16, 49]	*U* = 201.0	0.927
Sex (female)	5 (35.7%)	4 (26.7%)	χ^2^ = 0.02	0.700
Daily cigarettes	0 (0) [0, 45]	0 (4) [0, 27]	*U* = 220.0	0.304
War/torture events (vivo event list)	7.5 (11.7) [0, 19]	2.0 (6.0) [0, 20]	*U* = 191.5	0.140
Civil traumatic events (CAPS event list)	7.5 (2.5) [5, 13]	7.0 (4.5) [1, 15]	*U* = 201.5	0.300
Lifetime traumatic event exposure^a^	17.5 (12.2) [5, 29]	9.0 (10.5) [1, 24]	*U* = 185.5	0.084
PTSD symptom severity (CAPS)	81.5 (26.0) [63, 113]	17.5 (41.0) [0, 65]	*U* = 106.0	< 0.001
Depressive symptom severity (HAMD)	28.0 (14.0) [12, 44]	5.0 (14.0) [0, 31]	*U* = 114.5	0.002
Comorbid major depressive disorder	10 (71.4%)	5 (33.3%)	χ^2^ = 2.82	0.066
Somatic symptoms (SOMS-7)	21.0 (9.5) [5, 48]	8.0 (23.0) [0, 41]	*U* = 182.5	0.063
Chronic physical disease	3 (21.4%)	4 (28.6%^b^)	χ^2^ = 0	1

Values represent *Mdn* (*IQR*) [range] or *n* (%); *U* = Mann–Whitney *U* test; χ^2^ = Fisher’s exact test. All tests were calculated against α < 0.05 (two-tailed).

^a^Sum of CAPS and Vivo event checklists.

^b^One missing value.

The inclusion criterion for the PTSD group was a diagnosis of current PTSD according to DSM-IV-TR. Participants of the control group had no current PTSD. MDD was accepted as (comorbid) diagnosis in both groups (Table 1). Exclusion criteria for participation were psychotic disorders. Psychotropic medication was taken by two individuals with PTSD (hypnotics, antidepressants/neuroleptics) and one individual without PTSD (antidepressant).

The Ethics Committee of the University of Konstanz approved all study procedures. All participants received detailed information about the study aims and procedures before providing written informed consent. Participants received an allowance of €30.

### Clinical assessment

Trained psychologists specialized in trauma therapy conducted standardized diagnostic interviews with all the participants. Trained interpreters supported the communication if necessary. The participants’ general psychiatric status was assessed with the German Mini-International Neuropsychiatric Interview for DSM-IV^26^. Diagnosis and severity of PTSD as well as exposure to civil traumatic events were assessed with the German Clinician-Administered PTSD Scale (CAPS)^27^. Exposure to traumatic events in the context of war, displacement, detention, and torture was assessed using the vivo checklist^28^. The German 21-item Hamilton Depression Rating Scale (HAMD)^29^ and a shortened version of the screening for somatoform disorder (SOMS-7)^30^ served to quantify the severity of depressive and somatic symptoms, respectively.

### Blood sampling and PBMC isolation

Between 11:30 a.m. and 2:30 p.m., 30 ml venous blood was collected in an S-Monovette 10 ml 9NC (Sarstedt, Germany). PBMCs were isolated via density gradient centrifugation using Biocoll® separating solution (Biochrome AG, Germany). Briefly, blood was diluted with phosphate-buffered saline (PBS; Sigma-Aldrich, Germany) in a 1:1 concentration. 25 ml Blood-PBS solution was carefully pipetted into a 50 ml Falcon tube on top of 15 ml Biocoll® separating solution. After centrifugation (10 min, room temperature, 400 g, without brake), PBMC layer was harvested. Cells were washed twice in 40 ml PBS (10 min, 4 °C, 200 g). Two-thirds of the isolated PBMCs were pelleted again (5 min, 4 °C, 200 g), resuspended in RPMI-1640 medium (Invitrogen, USA) containing 10% fetal calf serum (FCS; Thermo Fisher Scientific, Germany), 100 U/ml penicillin (Invitrogen; USA), 100 mg/ml streptomycin (Invitrogen, USA) and 10% dimethyl sulfoxide (Sigma-Aldrich, Germany) at 2–10 × 10^6^ cells/ml. Cells were stored at − 80 °C prior to RNA extraction. One-third of the isolated PBMCs was pelleted again (5 min, 4 °C, 200 g) and resuspended in cold RPMI-1640 medium containing 10% FCS, 100 U/ml penicillin and 100 mg/ml streptomycin at 5 × 10^5^ cells/ml. Cells were kept on ice prior to DNA damage and repair analysis.

### Gene expression

Total RNA was isolated using the NucleoSpin® RNA II Isolation Kit (Macherey–Nagel, Germany) according to the manufacturer’s protocol. RNA was eluted in 30 µl DNA/RNase free H_2_O and the concentration was determined photometrically using Nanodrop spectrophotometer. RNA was transcribed into cDNA via Verso cDNA Kit (Thermo Fisher Scientific, Germany) and diluted to 5 ng/µl. For gene expression analyses, semi-quantitative polymerase-chain reaction was conducted. *ACTB* served as reference gene for quantification. 15 µl Red-Taq Ready Mix™ (Sigma-Aldrich, Germany), 4 µl cDNA, 1 µl forward primer, 1 µl reverse primer and 9 µl DNA/RNase free water was added in a 96 well PCR-plate. The following PCR program was used: 2 min 95 °C initial denaturation, 24 cycles of amplification: denaturation 30 s 95 °C, annealing 45 s 60 °C, elongation 1 min 72 °C, and terminal elongation 10 min 72 °C. PCR products were separated on a 1% agarose gel and stained with EtBr and imaged upon UV exposure. Signal intensity of target genes were normalized to signal intensity of *ACTB*. The *XRCC1* expression value of one case was excluded due to being unexpectedly high (9.7).

### DNA damage and repair kinetics

The automated version of the Fluorometric Detection of Alkaline DNA Unwinding (FADU) assay was used for assessment of basal DNA damage and repair kinetics in combination with prior induction of ex vivo DNA damage (mainly DNA-SSB) via X-ray irradiation as previously described^31,32^. Briefly, samples for baseline DNA damage (P0) were kept on ice, whereas DNA repair samples (R1–R9) and control samples without repair (PX) were irradiated on ice with 3.8 Gy using an X-ray generator (CHF Müller, Hamburg, Germany, 70 keV, 1 mm Al-filter). PX was immediately transferred on ice. To allow DNA repair, R1–R9 samples were incubated at 37 °C for 10 to 90 min. Afterwards, cells were lysed, and subsequently, DNA-strand breaks, sites of replication, and chromosome ends serve as starting points for DNA unwinding under alkaline conditions. DNA unwinding was stopped via neutralization of p*H*. Double-stranded DNA was stained with Sybr® Green and the fluorescence signal (485_ex_/535_em_) was detected at a fluorescence plate reader. Therefore, decrease in fluorescence intensity reflects an increase in DNA unwinding and, consequently, a higher number of DNA-strand breaks. FADU signals were log_10_-transformed. These data has already been reported elsewhere^19^.

### Statistical analysis

Statistical analyses were performed in R 3.6.1^33^. Due to nonnormal distributions, we used Mann–Whitney *U*-tests to compare groups and Spearman correlations to examine bivariate associations. DNA-repair kinetics were analyzed using linear mixed models using the *lme4* package^34^. The models predicted the DNA integrity as measured with the FADU assay by the fixed within factor ‘Repair Time’ (comprising the 11 phases of the FADU assay) and the fixed between factor ‘Gene Expression’ of DNA repair proteins, along with their interaction (Repair Time × Gene Expression). To reflect the repeated measures, we modeled random intercepts for each subject as a random effect^35^. Model residuals were normal distributed. *P*-values of *post-hoc* tests were adjusted via Benjamini–Hochberg procedure^36^.

### Ethical standards

The authors assert that all procedures contributing to this work comply with the ethical standards of the relevant national and institutional committees on human experimentation and with the Helsinki Declaration of 1975, as revised in 2008.

## Results

### Gene expression

#### XRCC1

**Figure 1:**
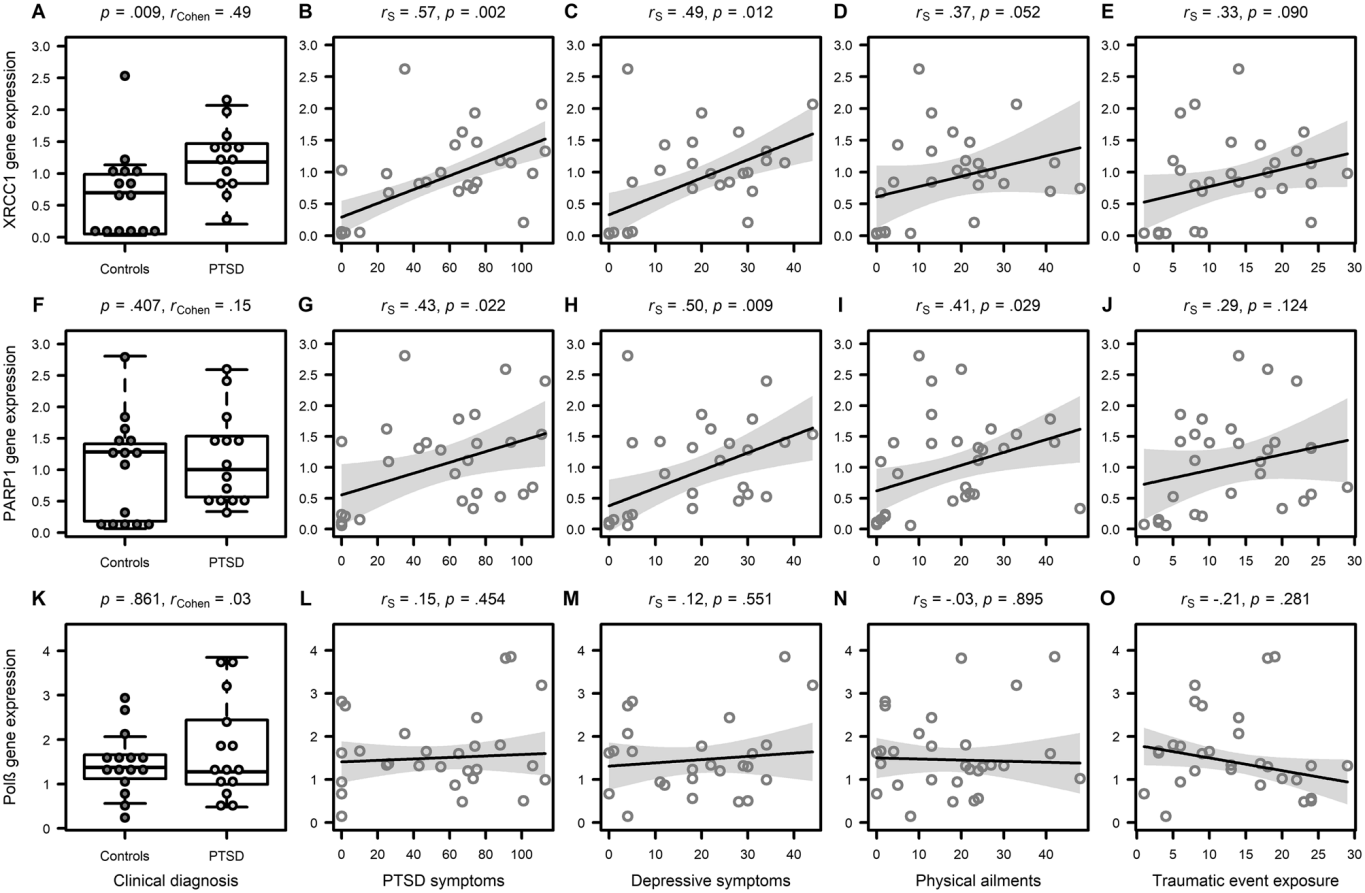
Association between DNA-repair gene expression and mental health status. Scatter plots displaying the estimated associations between expression of DNA-repair genes and mental health status. Results were obtained with Mann–Whitney *U*-tests for group comparisons and Spearman correlations (*r*_S_) for bivariate associations.

*XRCC1* gene expression was significantly higher in PBMCs of PTSD patients than controls (*U* = 161.0, *p* = 0.009w, Cohen’s *r* = 0.49). In the entire cohort, *XRCC1* gene expression was positively associated with PTSD symptoms (*r*_S_ = 0.57, *p* = 0.002) and depressive symptoms (*r*_S_ = 0.49, *p* = 0.012). In trend, *XRCC1* gene expression was descriptively higher in subjects with higher lifetime traumatic event exposure (*r*_S_ = 0.33, *p* = 0.090) and more somatic symptoms (*r*_S_ = 0.37, *p* = 0.052). Figure 1 displays the findings. Adjusting for age and smoking did not alter the results (Supplementary Table 1).

#### PARP1

*PARP1* gene expression in PBMCs did not differ between PTSD patients and controls (*U* = 206.0, *p* = 0.407, Cohen’s *r* = 0.15). However, in the whole cohort, *PARP1* gene expression was positively associated with PTSD symptoms (*r*_S_ = 0.43, *p* = 0.022), depressive symptoms (*r*_S_ = 0.50, *p* = 0.009), and somatic symptoms (*r*_S_ = 0.41, *p* = 0.029). There was no association between *PARP1* gene expression and lifetime traumatic event exposure (*r*_S_ = 0.29, *p* = 0.124). Figure 1 displays the findings. Adjusting for age and smoking did not alter the result pattern (Supplementary Table 2).

#### Polβ

*Polβ* gene expression in PBMCs did not differ between PTSD patients and controls (*U* = 221.0, *p* = 0.861, Cohen’s *r* = 0.03), and in the entire cohort, there were no associations with PTSD symptoms (*r*_S_ = 0.15, *p* = 0.454), depressive symptoms (*r*_S_ = 0.12, *p* = 0.551), somatic symptoms (*r*_S_ = -0.03, *p* = 0.895), and lifetime traumatic event exposure (*r*_S_ = − 0.21, *p* = 0.281). As *Polβ* gene expression was significantly lower with higher age (*r*_S_ = − 0.46, *p* = 0.013), we computed ANCOVAs to adjust for influences of age and daily cigarettes, which did not lead to different results (Supplementary Table 3). Figure 1 summarizes the findings.

### Functional relevance of altered gene expression

At baseline, DNA integrity was significantly lower in PBMCs of PTSD patients than controls (*U* = 200.0, *p* = 0.030, *r*_rb_ = 0.52). Descriptively, higher baseline DNA integrity was associated with lower gene expression of *XRCC1* (*r*_S_ = − 0.33, *p* = 0.112) and *PARP1* (*r*_S_ = − 0.29, *p* = 0.175), while there was no association with *Polβ* gene expression (*r*_S_ = − 0.05, *p* = 0.806).

We tested whether the gene expression of *XRCC1*, *PARP1*, and *Polβ* accounted for differences in DNA repair kinetics (see Table 2 for details). Linear mixed effect models revealed significant interactions of repair time × *XRCC1* gene expression (*p* = 0.010) and repair time × *PARP1* gene expression (*p* = 0.022). These interactions indicate the DNA repair kinetic after X-ray irradiation depended on the endogenous gene expression of *XRCC1* and *PARP1*, respectively.

**Table 2:** Results of linear mixed models.

	*F*	*df*_*1*_*, df*_*2*_	*p*	η_p_^2^	R^2^_m_ (R^2^_c_)
Fluorescence signals PX-R9	120.69	10, 218.01	< 0.001***	0.85	–
XRCC1 gene expression	7.63	1, 21.99	0.011*	0.26	–
Interaction	2.39	10, 218.00	0.010*	0.10	
Overall model	58.75	21, 215.66	< 0.001***	–	0.606 (0.892)
Fluorescence signals PX-R9	119.47	10, 218.00	< 0.001***	0.85	–
PARP1 gene expression	2.48	1, 22.00	0.129	0.10	–
Interaction	2.15	10, 218.01	0.022*	0.09	
Overall model	57.81	21, 215.66	< 0.001***	–	0.544 (0.891)
Fluorescence signals PX-R9	115.80	10, 218.01	< 0.001***	0.84	–
Polβ gene expression	1.20	1, 22.00	0.285	0.05	–
Interaction	1.41	10, 218.00	0.176	0.06	
Overall model	55.66	21, 215.66	< 0.001***	–	0.523 (0.888)

*Note*: Logarithmic (log_10_) fluorescence signals of the automated Fluorometric Detection of Alkaline DNA Unwinding (FADU) assay served as outcome.^*^ *p* < 0.050,^**^ *p* < 0.010,^***^ *p* < 0.001, two-tailed.

**Figure 2:**
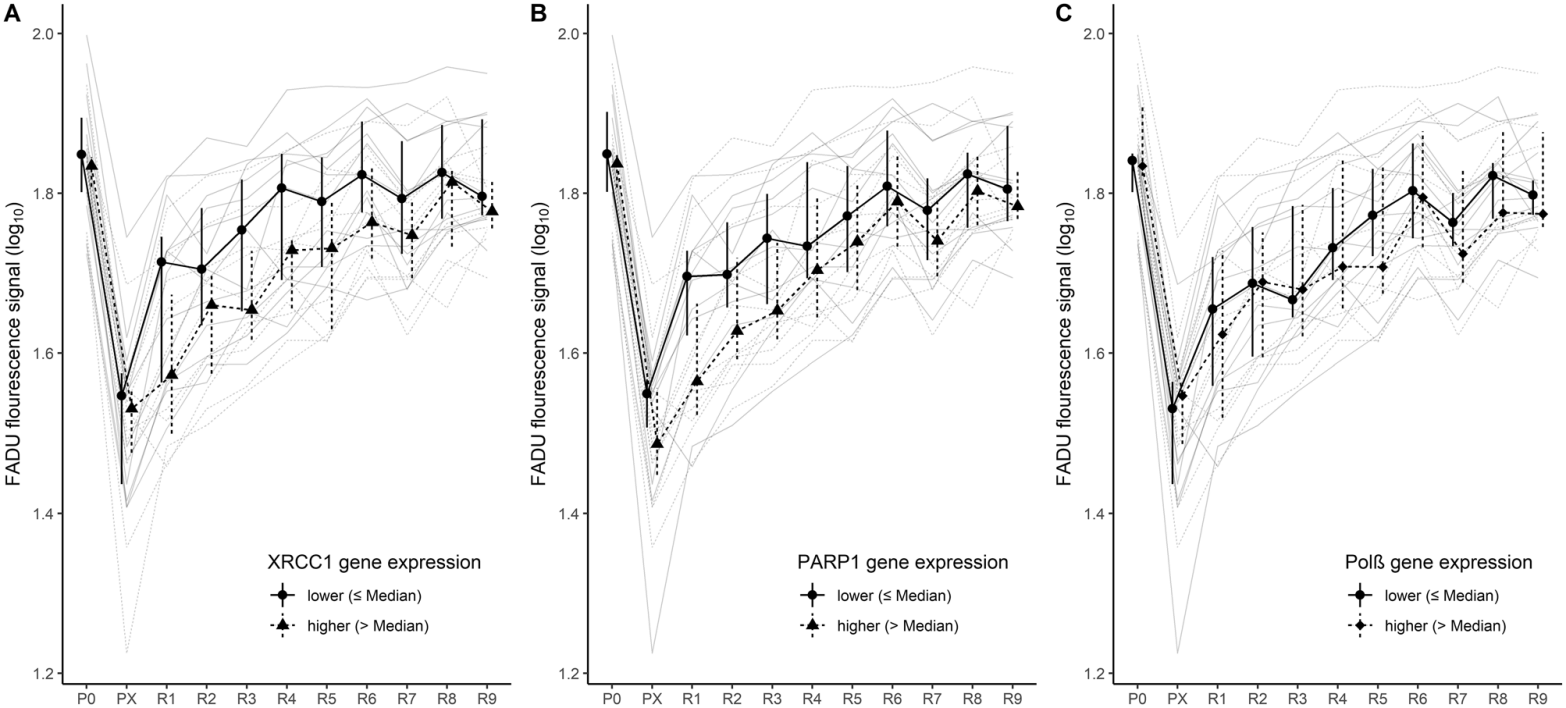
DNA integrity and repair kinetics as function of DNA-repair gene expression. Individual and averaged trajectories of DNA integrity at physiological levels (P0) and after X-ray irradiation (PX) as well as during repair for 10–90 min (R1–R9) as a function of (**A**) *XRCC1*, (**B**) *PARP1*, (**C**) *Polβ* gene expression. Averaged trajectories represent medians with error bars indicating the 1st and 3rd quartile. Gene expression have been analyzed as continuous variable, while for illustrating, the cohort has been divided in two arbitrary groups (lower vs. higher) by the median expression of the respective DNA-repair gene.

*Post-hoc* simple slopes (Supplementary Table 4) confirmed that higher *XRCC1* gene expression implicated lower fluorescence signals at PX to R6 (all *p*_FDR_’s ≤ 0.047), i.e., within the first 60 min of repair after X-ray irradiation. As Fig. 2A illustrates, PBMCs with *lower XRCC1* gene expression were *quicker* in repairing the DNA-SSB caused by X-ray irradiation.

Figure 2B suggests higher *PARP1* gene expression to implicate slower DNA repair within the first 20–30 min after X-ray irradiation. However, *post-hoc* simple slopes did not reveal significant effects of *PARP1* gene expression on the fluorescence signals (all *p*_FDR_’s ≥ 0.079).

There was no main or interaction effect of *Polβ* gene expression on FADU fluorescence signals (Table 2), indicating the gene expression of Polβ is not associated with DNA-SSB in PBMCs and does not account for differences in ex vivo DNA-repair kinetics (Fig. 2C).

Repeating the analyses while adjusting for age and smoking status did not alter the effects. Age and smoking did not significantly predict FADU fluorescence signals.

## Discussion

Traumatic stress and related mental disorders such as PTSD are associated with elevated levels of cellular oxidative stress as well as decreased anti-oxidative capacity^3,4,12–14^, altogether endangering DNA integrity through provoking genotoxic damage such as DNA-SSB. In line with this, we observed in our previous study^19^ that PBMCs from PTSD patients exhibit more DNA damage, namely DNA strand breaks, than PBMCs from controls without PTSD. As retaining genome integrity is essential for cellular survival, we hypothesized in our present study that immune cells of PTSD patients are required to increase their DNA maintenance and repair capabilities.

Indeed, our present study revealed that PBMCs from PTSD patients transcribe the *XRCC1* and conceivably the *PARP1* gene more frequently, suggesting that these cells seek to induce more DNA-repair proteins. However, changes in protein concentrations remain to be proven. No evidence for adaptations of the cells’ transcriptomic activity could be found for *Polβ*. Probably, PBMCs augment their DNA-repair capacity until reaching a physiologically tolerable equilibrium between enhanced repair kinetics and elevated oxidative damage dynamics. This compensatory effort might be interpreted as “allostatic load”^37^ on the cells: PBMCs from PTSD patients have to mobilize more effort in maintaining their DNA integrity than cells from non-PTSD controls. Our findings suggest elevated oxidative DNA damage as a biomolecular hallmark of traumatic stress and related psychopathology, with compensatory alterations in the expression of *XRCC1* and *PARP1* as novel biomarkers in traumatic stress and PTSD research.

To ascertain whether the observed changes in gene expression are of functional relevance for DNA integrity, we challenged the PBMCs’ potential to regain homeostasis through experimentally inducing severe amounts of DNA damage using ex vivo X-ray irradiation. PBMCs of all subjects were eventually able to restore their original DNA integrity within 90 min. However, PBMCs with higher gene expression of *XRCC1* and *PARP1* repaired DNA damage more slowly. We interpret this finding as demonstrating allostatic overload: increased expression of the *XRCC1* and *PARP1* genes might indicate ongoing efforts in compensating for pre-existing scarcity or higher demand for DNA-repair proteins. With additional damage as induced by our experiment, PBMCs facing pre-existing allostatic load are challenged to retain DNA integrity at less remaining reserve capacity. As a result, additional damage is repaired even more slowly. This preliminary interpretation needs further prove: As XRCC1, PARP1 and Polβ interact in a repair complex^38^, quantification of protein level as well as immunofluorescence experiments should be applied to reveal the actual amount of “occupied” repair proteins in cells from PTSD patients compared to healthy controls. Moreover, detection of PARylation would provide information about the activity of PARP1 and, therefore, quantify the amount of permanently ongoing DNA repair processes.

Through this study, we improved our understanding of trauma-related allostatic load in relation to DNA integrity, which is most likely mediated through oxidative stress and related DNA damage. Our findings point towards persistent alterations in cellular resource allocation in PTSD. Cellular resources may shift towards DNA maintenance and therefore might not be sufficiently available upon exposure to other cellular functions. For cells it is vital to prioritize repairing their DNA over other metabolic tasks. To this end, PARP1 is additionally regulating the cell cycle. It temporarily induces cell-cycle arrest during DNA repair that might have few consequences. However, in the long-run, persistent DNA repair and suspending the cell cycle provoke premature aging through senescence and apoptosis^22,39^. Consistently, increased numbers of immunosenescent T-cells^40^ and evidence for accelerated physiological aging was found in PTSD patients^41,42^.

PARP1 also mobilizes the transcription and translation of NF-κB, thus triggering the release of pro-inflammatory cytokines^22^. Indeed, there is meta-analytic evidence for modestly increased circulation of pro-inflammatory cytokines in individuals with chronic and traumatic stress as well as mental disorders such as PTSD^7^. As Del Giudice and Gangestad^43^ argue, the low-grade activity of pro-inflammatory cytokines is less an expression of actual systemic inflammation than a marker of ongoing repair and maintenance efforts of the body. This consideration derives further support by our observation of increased maintenance effort of DNA integrity in PTSD.

In addition, PARP1 utilizes NAD^+^ as a cofactor to fulfil its function of orchestrating the DNA-repair process^22^. Our previous ex vivo experiments demonstrated PARP1-related DNA repair crucially depends on NAD^+^ availability in PBMCs^44^. Additionally, PARP1-related NAD^+^ consumption upon DNA damage was shown to trigger a shift in cellular energy metabolism from mitochondrial oxidative phosphorylation towards glycolysis^45^. This energy-metabolic shift possibly serves to ensure NAD^+^ availability while at the same time decreasing additional oxidative stress-related DNA damage. In line with this, we and others have previously demonstrated alterations in the mitochondrial bioenergetics in traumatic stress and related mental disorders^46–48^.

The link between DNA maintenance, cell-cycle control, and energy metabolism is highlighting the complexity of the systemic and molecular mechanisms underlying the etiology of stress-related mental disorders^1,4,15^. Moreover, PTSD is associated with premature onset of various non-communicable degenerative diseases affecting the cardiovascular, respiratory, gastrointestinal, and immune system^1,2^. Our findings indicate that traumatic stress threatens DNA maintenance in immune cells and could thereby accelerate immunological aging. It remains to be investigated whether the physiological comorbidity pattern of PTSD results from permanently “straining” the organism’s DNA-repair capacity due to increased levels of oxidative stress.

### Limitations

This case–control study investigated three genes with central functions in retaining DNA integrity via BER. Our interpretation is essentially limited to gene expression of XRCC1 and PARP1, and DNA repair kinetics. Moreover, due to limited cell material we could not measure the actual protein level in PBMCs, and our interpretation of elevated gene expression as a marker of increased need for DNA-repair proteins remains to be confirmed. In addition, the BER of DNA comprises also other proteins. Future studies should combine transcriptomics and proteomics data (e.g., along with ingenuity pathway analysis) to characterize the activation of the entire BER system and relate it to functional assays of DNA repair. It should be noted that our results are based on laboratory techniques carried in 2014. Replications and further studies should use state-of-the-Art techniques (e.g. qPCR). To assess DNA-repair kinetics, we used ex vivo X-ray irradiation to elicit intense DNA damage, which may not necessarily reflect physiological conditions. Our participants encountered multiple adversities during displacement and flight; thus, their mental health burden was probably higher than amongst individuals with PTSD from the common population of Western industrialized countries. Accordingly, cohorts with less traumatic event exposure and/or lower symptom burden might show fewer stress correlates on cellular levels.

## Conclusions

Immune cells of PTSD patients exhibit increased physiological levels of DNA damage along with increased expression of certain genes involved in BER, which we demonstrated to be of functional relevance for DNA-repair kinetics. These findings should serve as a starting point for future studies on DNA integrity and the regulation of BER in PTSD using combined transcriptomics and proteomics approaches. This study highlights the systemic impact of psychological trauma, apparently straining PTSD patients’ genome maintenance. Moreover, our study points towards the contribution of oxidative DNA damage and allostatic processes in the immune system and cellular energy metabolism to physiological health decline after psychological stress. Future longitudinal studies are warranted to assess the reversibility of the observed allostatic load processes.

## Supplementary Information

Supplementary Tables.

## Data Availability

The dataset used and analyzed during the current study is available from the corresponding author upon reasonable request.
