# Supplementary material for: Expression of DNA repair genes and its relevance for DNA repair in peripheral immune cells of patients with posttraumatic stress disorder
Source: Sci Rep. 2022 Nov 4;12:18641. doi: 10.1038/s41598-022-22001-w (PMC9636148; doi:10.1038/s41598-022-22001-w)
Supplement: Supplementary file 1 — Supplementary Tables. [file 41598_2022_22001_MOESM1_ESM.docx]

Supplementary Table 1. Results of robust ANCOVAs with *XRCC1* expression as outcome

| **Model** | **Predictors** | ***b* (*SE*)** | ***F*** | ***df*s** | ***p*** | **η_p_²** |
| --- | --- | --- | --- | --- | --- | --- |
| A | Group | 0.70 (0.20) | 12.16 | 1, 23 | .002^**^ | .35 |
|  | Age | 0.00 (0.01) | 0.22 | 1, 23 | .644 | .01 |
|  | Daily cigarettes | -0.01 (0.02) | 0.30 | 1, 23 | .590 | .01 |
| B | PTSD symptom severity | 0.01 (0.00) | 36.28 | 1, 23 | < .001^***^ | .61 |
|  | Age | -0.01 (0.01) | 0.97 | 1, 23 | .644 | .04 |
|  | Daily cigarettes | 0.02 (0.01) | 1.63 | 1, 23 | .215 | .07 |
| C | Lifetime traumatic event exposure | 0.04 (0.02) | 6.68 | 1, 23 | .017^*^ | .23 |
|  | Age | -0.01 (0.01) | 1.41 | 1, 23 | .248 | .06 |
|  | Daily cigarettes | -0.02 (0.01) | 7.20 | 1, 23 | .013^*^ | .24 |
| D | Depressive symptom severity | 0.03 (0.01) | 30.08 | 1, 21 | < .001^***^ | .59 |
|  | Age | 0.00 (0.01) | 0.02 | 1, 21 | .881 | .00 |
|  | Daily cigarettes | -0.02 (0.01) | 5.74 | 1, 21 | .026^*^ | .21 |
| E | Severity of physical ailments | 0.02 (0.01) | 3.16 | 1, 23 | .089 | .12 |
|  | Age | -0.01 (0.01) | 0.99 | 1, 23 | .330 | .04 |
|  | Daily cigarettes | -0.01 (0.01) | 1.57 | 1, 23 | .223 | .06 |

*Note*: Overall model statistics of model A: *F*(3, 23) = 4.57, *p* = .012, R² = .333; B: *F*(3, 23) = 14.63, *p* < .001, R² = .634; C: *F*(3, 23) = 4.35, *p* = .014, R² = .202; D: *F*(3, 21) = 13.85, *p* < .001, R² = .574; E: *F*(3, 23) = 1.69, *p* = .197, R² = .159. ^*^ *p* < .050, ^**^ *p* < .010, ^***^ *p* < .001, two-tailed.

Supplementary Table 2. Results of robust ANCOVAs with *PARP1* expression as outcome

| **Model** | **Predictors** | ***b* (*SE*)** | ***F*** | ***df*s** | ***p*** | **η_p_²** |
| --- | --- | --- | --- | --- | --- | --- |
| A | Group | 0.18 (0.30) | 0.35 | 1, 24 | .558 | .01 |
|  | Age | -0.02 (0.02) | 1.78 | 1, 24 | .194 | .07 |
|  | Daily cigarettes | -0.01 (0.01) | 0.77 | 1, 24 | .389 | .03 |
| B | PTSD symptom severity | 0.01 (0.00) | 5.79 | 1, 24 | .024^*^ | .19 |
|  | Age | -0.01 (0.02) | 0.91 | 1, 24 | .349 | .04 |
|  | Daily cigarettes | -0.01 (0.01) | 1.55 | 1, 24 | .225 | .06 |
| C | Lifetime traumatic event exposure | 0.04 (0.02) | 2.86 | 1, 24 | .104 | .11 |
|  | Age | -0.02 (0.01) | 2.57 | 1, 24 | .122 | .10 |
|  | Daily cigarettes | -0.02 (0.01) | 3.42 | 1, 24 | .077 | .12 |
| D | Depressive symptom severity | 0.03 (0.01) | 10.10 | 1, 21 | .004^**^ | .32 |
|  | Age | -0.02 (0.01) | 1.17 | 1, 21 | .291 | .05 |
|  | Daily cigarettes | -0.02 (0.01) | 11.25 | 1, 21 | .003^**^ | .35 |
| E | Severity of physical ailments | 0.02 (0.01) | 3.00 | 1, 24 | .096 | .11 |
|  | Age | -0.02 (0.01) | 2.95 | 1, 24 | .099 | .11 |
|  | Daily cigarettes | -0.01 (0.01) | 1.89 | 1, 24 | .182 | .07 |

*Note*: Overall model statistics of model A: *F*(3, 24) = 0.78, *p* = .517, R² = .078; B: *F*(3, 24) = 2.55, *p* = .079, R² = .243; C: *F*(3, 24) = 2.86, *p* = .058, R² = .183; D: *F*(3, 21) = 8.13, *p* < .001, R² = .418; E: *F*(3, 24) = 2.45, *p* = .088, R² = .203. ^*^ *p* < .050, ^**^ *p* < .010, two-tailed.

Supplementary Table 3. Results of robust ANCOVAs with *Polymerase β* expression as outcome

| **Model** | **Predictors** | ***b* (*SE*)** | ***F*** | ***df*s** | ***p*** | **η_p_²** |
| --- | --- | --- | --- | --- | --- | --- |
| A | Group | -0.20 (0.26) | 0.61 | 1, 24 | .443 | .02 |
|  | Age | -0.04 (0.01) | 7.38 | 1, 24 | .012^*^ | .24 |
|  | Daily cigarettes | -0.02 (0.01) | 3.11 | 1, 24 | .091 | .11 |
| B | PTSD symptom severity | 0.00 (0.01) | 0.07 | 1, 24 | .794 | .00 |
|  | Age | -0.04 (0.01) | 7.63 | 1, 24 | .011^*^ | .24 |
|  | Daily cigarettes | -0.02 (0.01) | 2.24 | 1, 24 | .147 | .09 |
| C | Lifetime traumatic event exposure | -0.01 (0.01) | 0.53 | 1, 24 | .473 | .02 |
|  | Age | -0.04 (0.02) | 5.34 | 1, 24 | .030^*^ | .18 |
|  | Daily cigarettes | -0.02 (0.01) | 1.88 | 1, 24 | .183 | .07 |
| D | Depressive symptom severity | -0.01 (0.01) | 1.44 | 1, 21 | .243 | .06 |
|  | Age | -0.06 (0.01) | 16.36 | 1, 21 | < .001^***^ | .44 |
|  | Daily cigarettes | -0.03 (0.01) | 17.99 | 1, 21 | < .001^***^ | .46 |
| E | Severity of physical ailments | -0.01 (0.01) | 0.68 | 1, 24 | .418 | .03 |
|  | Age | -0.04 (0.02) | 5.54 | 1, 24 | .027^*^ | .19 |
|  | Daily cigarettes | -0.02 (0.01) | 2.62 | 1, 24 | .119 | .10 |

*Note*: Overall model statistics of model A: *F*(3, 24) = 3.93, *p* = .020, R² = .310; B: *F*(3, 24) = 2.63, *p* = .073, R² = .288; C: *F*(3, 24) = 1.80, *p* = .174, R² = .373; D: *F*(3, 21) = 14.11, *p* < .001, R² = .521; E: *F*(3, 24) = 2.00, *p* = .141, R² = .326. ^*^ *p* < .050, ^**^ *p* < .010, ^***^ *p* < .001, two-tailed.

Supplementary Table 4. Post-hoc simple slopes for the *XRCC1* model

| **FADU sample** | ***b*** | ***SE*** | ***df*** | ***t-r*atio** | ***p*_FDR_** |
| --- | --- | --- | --- | --- | --- |
| P0 | -0.03 | 0.02 | 38.61 | -1.46 | .166 |
| PX | -0.06 | 0.02 | 38.61 | -2.52 | .034^*^ |
| R1 | -0.09 | 0.02 | 38.69 | -3.97 | .003^**^ |
| R2 | -0.07 | 0.02 | 38.61 | -2.87 | .025^*^ |
| R3 | -0.08 | 0.02 | 38.61 | -3.20 | .015^*^ |
| R4 | -0.06 | 0.02 | 38.61 | -2.73 | .026^*^ |
| R5 | -0.06 | 0.02 | 38.61 | -2.46 | .034^*^ |
| R6 | -0.05 | 0.02 | 38.61 | -2.25 | .047^*^ |
| R7 | -0.04 | 0.02 | 38.61 | -1.89 | .082 |
| R8 | -0.04 | 0.02 | 38.61 | -1.88 | .082 |
| R9 | -0.03 | 0.02 | 38.69 | -1.09 | .281 |

*Note*: The automated Fluorometric Detection of Alkaline DNA Unwinding (FADU) assay analyzes several samples, of which P0 reflects the physiological level of DNA damage; PX reflects the DNA damage after X-ray irradiation; R1–R9 reflect the DNA damage after 10 to 90 min of repair at 37 °C in 10 min intervals. ^*^ *p* < .050, ^**^ *p* < .010, two-tailed, corrected with false discovery rate (FDR).
